# PRELIMINARY RESEARCH ON TOUCHPOINT METHOD TO ENGAGE CHINESE OLDER ADULTS IN PARTICIPATORY DESIGN ACTIVITIES

**DOI:** 10.1093/geroni/igae098.3184

**Published:** 2024-12-31

**Authors:** Jiabei Jiang, Tongxin Sun, Jianing Yin, Yuanyuan Liu

**Affiliations:** Nanjing university of the arts, Nanjing, Jiangsu, China (People’s Republic); Tsinghua University, Beijing, Beijing, China (People’s Republic); Tsinghua University, Beijing, Beijing, China (People’s Republic); School of Mechanical Engineering & Automation, Beihang University, Beijing, Beijing, China (People’s Republic)

## Abstract

Collaborative engagement with older adults in design research activities is an effective and beneficial method for researchers to understand the needs of this demographic. Participatory design, originally proposed within Western value systems, faces challenges in application across different regions and cultural contexts, particularly in participatory design activities targeting older adults within the Chinese societal context. However, limited literature focuses on the methodologies of participatory design practices with older adults in non-Western countries. This study views the organizational process of participatory design activities as a form of service design research involving older adults, where design researchers serve as providers and older adults as the recipients of the service. Drawing upon service design thinking, this paper introduces the concept of ‘participation touchpoints’—moments within activities that encourage active involvement of older adults—to explore the interactions between participants and the organizers, procedures, and environments of activities. This approach aims to optimize the experience of older participants and enhance their engagement. Furthermore, based on the Theory of Planned Behavior, we proposed a framework of participation touchpoints including attitude guidance, incentive motivation, and capability compensation. We evaluated this framework through a participatory design workshop with older adults and multiple stakeholders, focusing on the dynamic interactions among organizers, participants, and the activities themselves. This research endeavors to foster more effective collaboration with older adults in design research, aiming to develop a participatory design framework that ensures mutual empowerment for older adults and the research team.

